# Analysis of mechanical characteristics of walking and running foot functional units based on non-negative matrix factorization

**DOI:** 10.3389/fbioe.2023.1201421

**Published:** 2023-07-21

**Authors:** Xiaotian Bai, Hongfeng Huo, Jingmin Liu

**Affiliations:** ^1^ Department of Physical Education, Tsinghua University, Beijing, China; ^2^ College of Physical Education, Hebei Normal University, Shijiazhuang, China; ^3^ Key Laboratory of Bioinformatics Evaluation of Human Movement, Hebei Normal University, Shijiazhuang, China

**Keywords:** non-negative matrix factorization, one-dimensional statistical parameter mapping, foot pressure, walking, running

## Abstract

**Objective:** To explore the characteristics of Non-Negative Matrix Factorization (NNMF) in analyzing the mechanical characteristics of foot functional units during walking and running.

**Methods:** Eighteen subjects (9 males and 9 females) were recruited, and the ground reaction force curves of each foot region during walking and running were collected using a plantar pressure measurement system. NNMF was used to extract the mechanical features of different foot regions and to determine the number of foot functional units. The differences between the base matrices of walking and running were compared by traditional t-tests, and the differences in coefficient matrices were compared by one-dimensional statistical parameter mapping.

**Results:** 1) When the number of foot functional units for walking and running were both 2, the Variability Accounted For (VAF) by the matrix exceeded 0.90 (VAF _walk_ = 0.96 ± 0.02, VAF _run_ = 0.95 ± 0.04); 2) In foot functional unit 1, both walking and running exhibited buffering function, with the heel region being the main force-bearing area and the forefoot also participating in partial buffering; 3) In foot functional unit 2, both walking and running exhibited push-off function, with the middle part of the forefoot having a higher contribution weight; 4) In foot functional unit 1, compared to walking, the overall force characteristics of the running foot were greater during the support phase of the 0%–20% stage, with the third and fourth metatarsal areas having higher contribution weights and the lateral heel area having lower weights; 5) In foot functional unit 2, compared to walking, the overall force was higher during the beginning and 11%–69% stages of running, and lower during the 4%–5% and 73%–92% stages. During running, the thumb area, the first metatarsal area and the midfoot area had higher contribution weights than during walking; in the third and fourth metatarsal areas, the contribution weights were lower during running than during walking.

**Conclusion:** Based on the mechanical characteristics of the foot, walking and running can both be decomposed into two foot functional units: buffering and push-off. The forefoot occupies a certain weight in both buffering and push-off functions, indicating that there may be a complex foot function transformation mechanism in the transverse arch of foot. Compared to walking, running completes push-off earlier, and the force region is more inclined towards the inner side of the foot, with the hallux area having a greater weight during push-off. This study suggests that NNMF is feasible for analyzing foot mechanical characteristics.

## 1 Introduction

Walking and running are the most basic forms of human movement. The low cost and convenience make brisk walking and slow running a popular way of exercising (Wolf and Wohlfart, 2014). In the process of walking and running, the foot as the supporting part of the human body not only bears the weight of the body but also absorbs and releases energy, cushions ground impact, and completes extension (Natali et al., 2010; Hageman et al., 2011; Kelly et al., 2014). Therefore, functional analysis of the foot during walking and running has important value. Foot pressure is the most commonly used analysis index to reflect foot function. Measuring foot pressure can help understand the support method and pressure distribution of the foot., evaluate the balance, stability and motion control ability of the foot (Hu et al., 2023; Jia et al., 2023). At present, foot pressure testing and analysis have been applied to rehabilitation programs and sports training program development (Wikstrom and McKeon, 2014; Jiang et al., 2021; Conner et al., 2022), foot function evaluation (Hillstrom et al., 2013; Montagnani et al., 2021; Shen et al., 2021), and sports shoe research and development (Chen et al., 1994; Zhao and Li, 2019).

Currently, assessment of plantar pressure usually divides the foot into four major zones: toe zone, metatarsal zone, midfoot zone, and heel zone, and each zone can be further divided into different subzones based on anatomical features. Functional analysis of the foot is performed using metrics such as impulse, maximum pressure, maximum pressure intensity and force loading rate in different regions of the foot (Aminian et al., 2013; Hillstrom et al., 2013). However, the structure and function of the foot are often closely related, and it is insufficient to divide the plantar area based solely on anatomical features, which may overlook the same mechanical properties of different regions of the foot, resulting in redundant regional divisions. At the same time, traditional plantar pressure indicators cannot simultaneously consider the spatiotemporal characteristics of foot mechanics. Therefore, from the perspective of functional analysis of foot movement, the shortcomings of traditional plantar pressure analysis methods can lead to information loss in the data analysis process, making it difficult to explore the dynamic mechanical characteristics of the foot.

Non-Negative Matrix Factorization (NNMF) is a data analysis method used to process complex non-negative data. It completes dimensionality reduction by decomposing the original matrix into a base matrix that reflects the weight of each element and a coefficient matrix that reflects the overall characteristics of each element. This makes the extracted data have clustering and temporal characteristics (Lee and Seung, 1999). Existing research in sports science mostly applies NNMF to analyze Surface Electromyographic Signals (sEMG) to explore muscle coordination characteristics of complex human action control mechanisms and action learning rules (Hagio et al., 2015; Hajiloo et al., 2020; Bach et al., 2021). In the medical field, NNMF can be used to predict the potential relationship between drugs and diseases, or detect the distribution of tumors based on magnetic resonance imaging (Laudadioa et al., 2016; Wang et al., 2022). In addition, NNMF can also be used for signal processing applications such as audio separation and feature extraction (O'Grady and Pearlmutter, 2008; Li et al., 2017). As plantar pressure has non-negative characteristics, some researchers have applied this method to functional partitioning of the foot and it has been proven to be feasible (Van Hese et al., 2021). Although NNMF can be combined with motion forms to divide foot functional areas, traditional statistical methods still cannot meet the requirements of curve analysis because the coefficient matrix of NNMF is a continuous data model. One-Dimensional Statistical Parameter Mapping (SPM1D) based on random field theory can be used for topological analysis of continuous data models, which has been applied in one-dimensional biomechanical data such as torque and angle (Pataky, 2012; Pataky et al., 2013; Pataky et al., 2015). Based on the research of Van Hese et al. (Van Hese et al., 2021), this paper incorporates SPM1D to the traditional statistical method to achieve quantitative analysis of the decomposed matrix by NNMF.

Combining the above content, this study collected plantar pressure data during walking and running and applied NNMF and SPM1D methods to divide the foot according to the mechanical characteristics of walking and running, extract foot functional units, and compare the dynamic foot mechanical characteristics of walking and running. This provides methodological support and theoretical support for the simplified processing of complex foot mechanical data and dynamic mechanical feature comparison of the foot.

## 2 Materials and methods

### 2.1 Participants

According to the sample size estimation using PASS software (Version15, NCSS, United States) for paired sample experimental design, when the significance level is 0.05, statistical power is 0.80, and effect size is 0.80, the minimum required sample size is 15. Based on the sample size estimation results, our study recruited 18 young participants for walking and running plantar pressure tests, including 9 male participants (age 25.2 ± 3.2 years, height 176.2 ± 4.3 cm, weight 68.3 ± 5.7 kg) and 9 female participants (age 24.3 ± 4.1 years, height 161.3 ± 3.7 cm, weight 51.9 ± 7.2 kg).

The participants involved in this experiment were normal foot types [with a foot arch index between 0.21–0.26 (Cavanagh and Rodgers, 1987)], All participants were healthy and had no history of lower limb surgery or lower limb injury within the past 3 months. They did not engage in strenuous exercise 48 h before the test. The dominant foot of the subjects was the right foot, and they ran with a rearfoot strike pattern. All subjects voluntarily participated in this experiment and signed an informed consent form. This experiment was approved by the Ethics Committee of Hebei Normal University (No. 2022LLSC026).

### 2.2 Experimental procedure and data acquisitions

**FIGURE 1:**
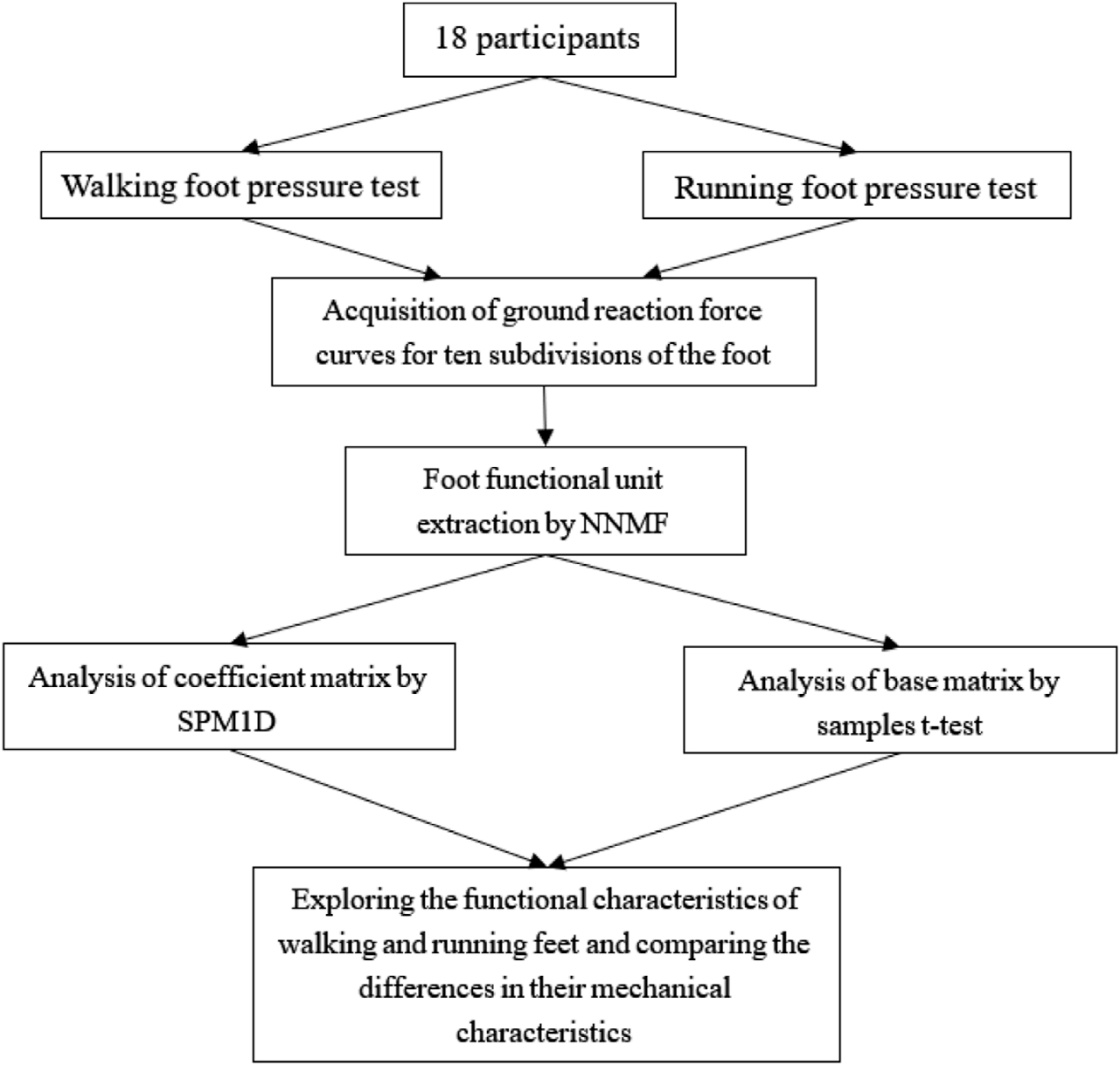
Technology roadmap.

Before the test began, the subjects were first familiarized with the experimental procedure under the explanation of the experimenter and completed warm-up under the guidance of the experimenter. After collecting basic information such as height, age, and weight, the subjects completed three barefoot walking and running tests in random order by drawing lots in a foot pressure test system with a length of 2 m and an extension runway of 1.5 m at both the starting point and the end point. According to previous research on the most suitable walking speed for young people (Lian et al., 2012; Baroudi et al., 2022), a speed closest to 1.50 m/s was recorded for the walking test, and the final walking speed range was 1.49 ± 0.09 m/s; in the running test, participants were selected to run at a speed close to twice walking speed (3.00 m/s) for recording purposes (Aghabayk et al., 2021), and the final running speed range was 3.08 ± 0.29 m/s. The Footscan high-frequency foot pressure test board produced by RSscan International Company was used in the test. The sampling frequency of the instrument was 126 Hz, the sensor density was 4 per square centimeter, the effective pressure measurement range was [1,60] N per square centimeter, and the minimum resolution was 0.25N. The technology roadmap of this study was shown in Figure 1.

### 2.3 Data processing

#### 2.3.1 Foot pressure data preprocessing

**FIGURE 2:**
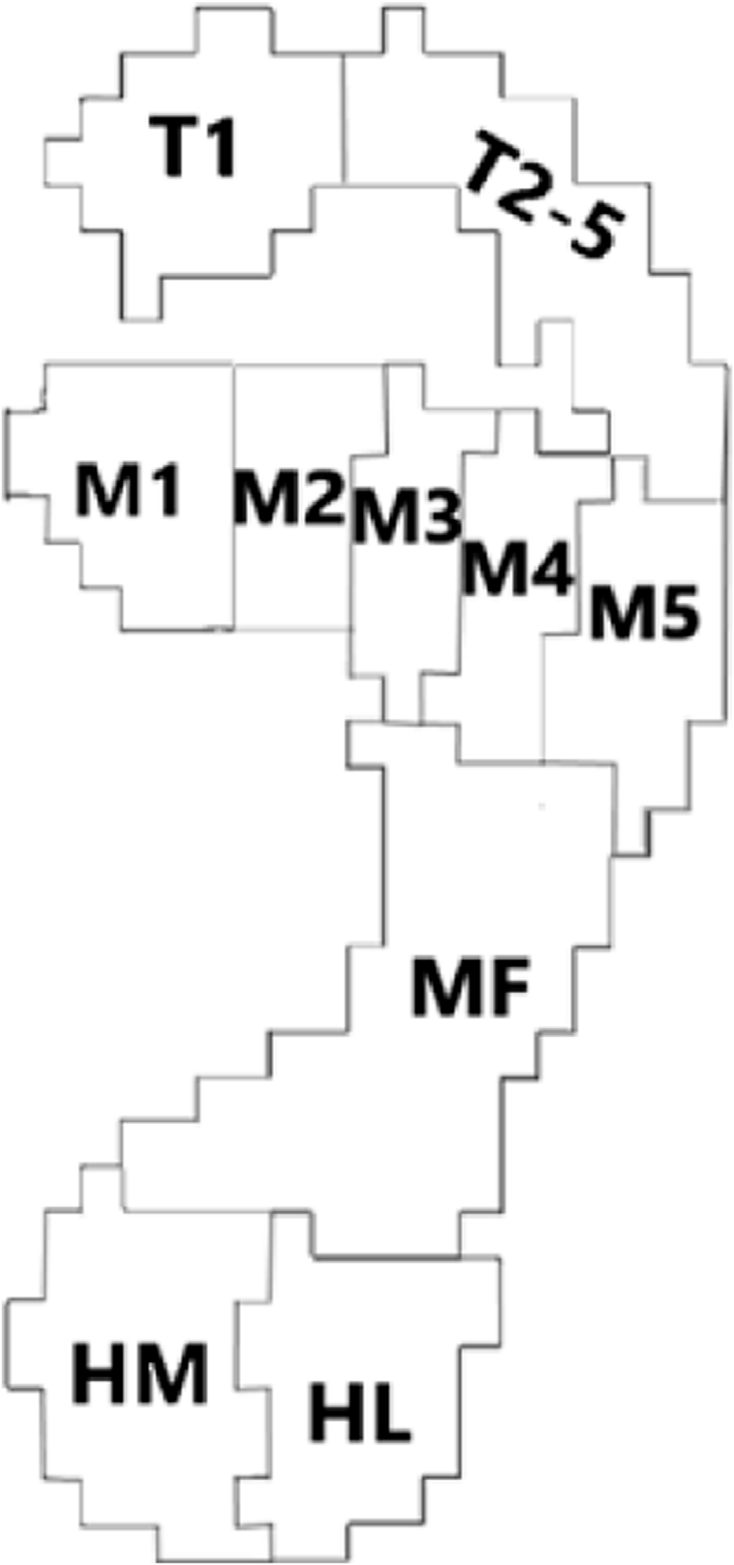
Foot partition schematic diagram.

Plantar pressure were divided into ten regions according to Footscan software (as shown in Figure 2), and the vertical ground reaction force changes of each region during walking and running support phases were collected. The collected foot pressure raw data of the ten regions were normalized according to the subject’s weight, where the acceleration due to gravity was taken as 9.8 m/s. The weight-normalized foot pressure data were filtered in Origin2018 using a fourth-order Butterworth low-pass filter with the cutoff frequency set to 20 Hz (Limroongreungrat and Boonkerd, 2019), and the curve was interpolated using cubic B-Spline (Warmenhoven et al., 2018). The number of points after interpolation was set to 101, which normalizes the support phase to an interval of 0%–100% (Warmenhoven et al., 2018).

(Note: Where T1 represents the thumb area, T2-5 represents the second to fifth toe area, M1 represents the first metatarsal area, M2 represents the second metatarsal area, M3 represents the third metatarsal area, M4 represents the fourth metatarsal area, M5 represents the fifth metatarsal area, MF represents the midfoot area, HM represents the medial heel area, and HL represents the lateral heel area.).

#### 2.3.2 Foot function unit extraction

The preprocessed plantar pressure data was arranged into a 10 row and 101 column matrix V based on 10 foot regions and 101 sampling points. NNMF was used to decompose matrix V into a 10-row k-column basis matrix W and a k-row 101-column coefficient matrix H, where k is the number of factors used in matrix decomposition (Lee and Seung, 1999) and represents the number of foot function units extracted (see Eq. 1). In order to eliminate the influence of random initialization matrix on the accuracy of results, NNMF was performed 10 times for each data, and the maximum VAF was extracted (Nishida et al., 2017). The matrix calculation was performed using the NNMF function combined with a loop statement in MATLAB 2022b. (Mathworks, USA), and the number of iterations was set to 5000 (Nishida et al., 2017; Sun, 2019).

$$\left[\begin{array}{cccc} V_{1\;1} & V_{1\;2} & \ldots & V_{1\;101}\\ V_{2\;1} & V_{2\;2} & \ldots & V_{2\;101}\\ \vdots & \vdots & \ldots & \vdots \\ V_{10\;1} & V_{10\;2} & \ldots & V_{10\;101} \end{array}\right]\approx \left[\begin{array}{ccc} W_{1\;1} & \ldots & W_{1\;k}\\ W_{2\;1} & \ldots & W_{2\;k}\\ \vdots & \ldots & \vdots \\ W_{10\;1} & \ldots & W_{10\;k} \end{array}\right]\times \left[\begin{array}{cccc} H_{1\;1} & H_{1\;2} & \ldots & H_{1\;101}\\ \vdots & \vdots & \ldots & \vdots \\ H_{k\;1} & H_{k\;2} & \ldots & W_{k\;101} \end{array}\right]$$ (1)

The Variability Accounted For (VAF) was used to evaluate the quality of the reconstructed matrix after decomposition. The VAF value ranges from 0 to 1, and the larger value of VAF indicates the higher quality of matrix reconstruction (Lee and Seung, 1999; Hug et al., 2010). In this study, the final number of foot functional units was determined when the VAF was greater than 0.85 and the change in VAF did not exceed 0.05 as the number of factors k increased, the convergence tolerance (TolFun) was set to 10^–6^ (Rabbi et al., 2020; Van Hese et al., 2021). The VAF calculation method is shown in (2):

$$VAF=1-\frac{RSS}{TSS}=1-\frac{\sum {\left(V_{mn}-V_{mn}^{\prime }\right)}^{2}}{\sum {V_{mn}}^{2}}$$ (2)

Where *RSS* is the residual sum of squares between the original matrix and the reconstructed matrix, *TSS* is the total sum of squares of the original matrix, $V_{mn}$ is the original matrix, and $V_{mn}^{\prime }$ is the reconstructed matrix.

As the matrices extracted from the same sport appear in a random order, it is necessary to classify the foot functional units. In this study, the cosine similarity was used to classify the units based on the curve characteristics of the coefficient matrix, the coefficient matrix in the extracted foot functional units from different subjects was compared with the initial coefficient matrix to rank the cosine similarity and thus classify the extracted foot functional units (Sy et al., 2016; Ghislieri et al., 2020; Van Hese et al., 2021). The cosine similarity is calculated as follows (3):

$${\cos\theta }_{i,k,n}=\frac{H_{i,k}\cdot H_{1,1}}{\left\|H_{i,k}\right\|\cdot \left\|H_{1,1}\right\|}$$ (3)

Where $H_{i,k}$ represents the k-th foot functional unit extracted from the i-th participant, and $H_{1,1}$ represents the initial coefficient matrix, and ${\cos\theta }_{i,k,n}$ represents the cosine similarity between $H_{i,k}$ and $H_{1,1}$ .

In the two decomposed matrices, the coefficient matrix H was used to evaluate the overall force characteristics of the foot during the support phase, and the basis matrix W was used to reflect the contribution of each foot region to the overall force characteristics of each foot functional unit. To unify the quantification standard, the weight of each foot region in each foot functional unit in the basis matrix W was normalized (Cappellini et al., 2006; Nishida et al., 2017), as shown in Eq. 4:

$$C_{wi}=\frac{1}{10}\sum_{n=1}^{10}C_{in}$$ (4)

Where *i* represents different foot functional units, *n* represents various foot regions, and $C_{wi}$ is the normalized weight of each foot region in each foot functional unit.

### 2.4 Data statistics

#### 2.4.1 Statistical test of coefficient matrix

For the coefficient matrix obtained by NNMF, SPM1D of paired experimental design was used to compare the force characteristics of walking and running foot functional units. When the curve residual value conforms to a normal distribution, the SPM1D model was used for testing. Otherwise, the SnPM1D was used for non-parametric model testing. The significance level α was set to 0.05, and the t-value calculation method of SPM/SnPM is as follows (Pataky et al., 2015):

$${SPM/SnPM}_{\left\{t\right\}}=\frac{\mu_{d}}{s_{d}/\sqrt{n}}$$ (5)

Where $\mu_{d}$ is the mean difference of the paired sample, $s_{d}$ is the standard deviation of the mean difference of the paired sample, and *n* is the number of paired samples.

According to the α value set in this study, the threshold $t_{critical}$ is calculated through random field theory. When $SPM/{SnPM}_{\left\{t\right\}}$ exceeds the threshold $t_{critical}$ , it is judged that there is statistical significance between the samples (Pataky et al., 2013; Pataky et al., 2015). The Eq. 5.

$$P\left(SPM/{SnPM}_{\left\{t\right\}}>t_{critical}\right)=1-\exp\left(-\int_{t_{critical}}^{\infty }f_{0D}\left(x\right)dx-ED\right)=\alpha$$ (6)

Where $f_{0D}\left(x\right)$ is the probability density function of the *t*-test; *ED* is the Euler density function related to smoothing.

#### 2.4.2 Statistical test of basic matrix

For the basic matrix obtained by NNMF, the weights of each plantar region were normalized and perform a normality test at first. If the data conforms to normal distribution, paired sample *t*-test was used for comparing the difference between walking and running basic matrices; if the data does not conform to normal distribution, Wilcoxon signed-rank test was used for statistics. When *p* < 0.05, indicating that the difference is significant.

## 3 Results

### 3.1 Number of foot functional units determined

#### 3.1.1 Determination of foot functional units for walking and running

**FIGURE 3:**
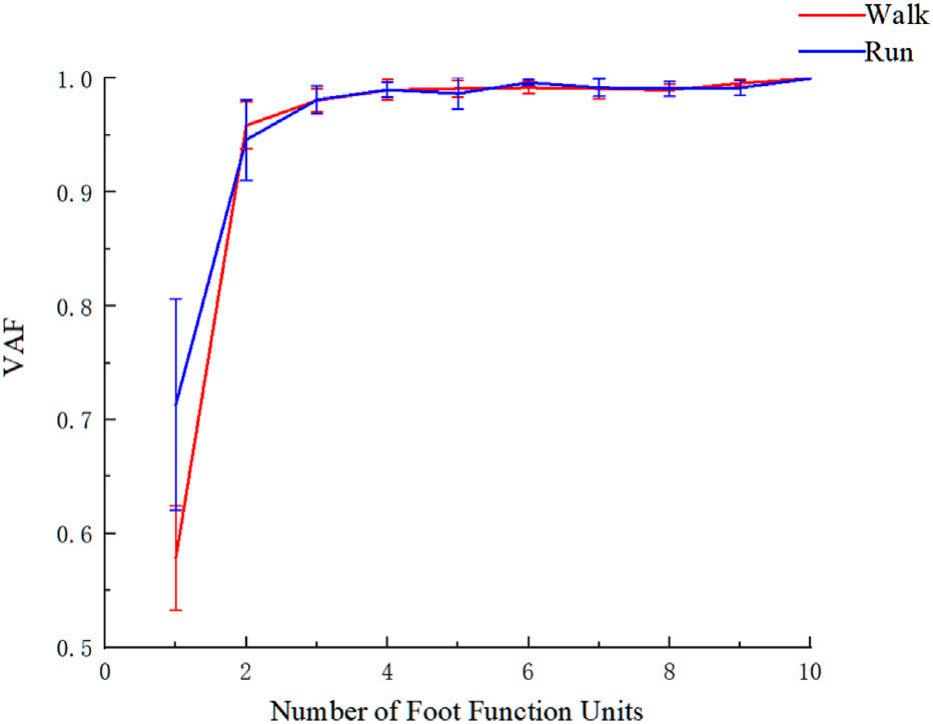
VAF vs. number of foot functional unit curve during walking and running.

Based on Figure 3, it can be observed that when the number of foot functional units is 2, the VAF of the reconstruction matrix is around 0.90 in both walking and running (with the VAF of 0.96 ± 0.02 for walking and 0.95 ± 0.04 for running), and the VAF does not significantly change when the number of foot functional units is greater than 2. Therefore, 2 foot functional units were selected for analysis during both walking and running.

### 3.2 Characteristics of foot functional units during walking and running

#### 3.2.1 Characteristics of foot functional units during walking

**FIGURE 4:**
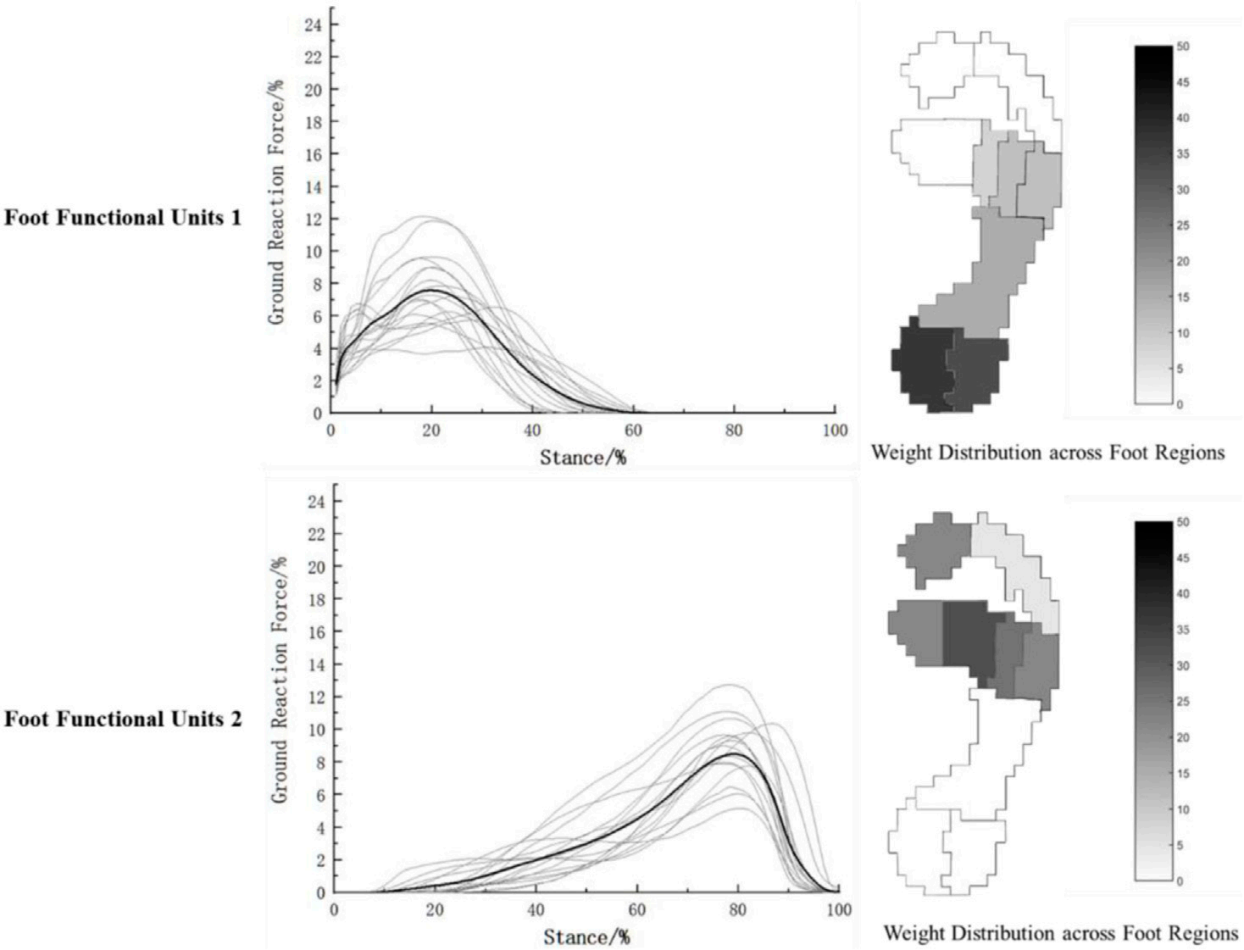
Characteristics of foot functional units during walking.

Based on the foot force characteristics during walking, the two foot functional units obtained through NNMF are shown in Figure 4, which respectively reflect the overall force characteristics (coefficient matrix) and the weight distribution across different foot regions (base matrix) of the two functional units. It can be observed from Figure 4 that during walking, the two foot functional units are dominated by the heel region and the mid-forefoot region consisting of the second, third, and fourth metatarsal bones, respectively. The overall force characteristics are concentrated around 20% and 80% of the stance phase.

(The gray lines show the foot force characteristics of each participant during walking, and the black line represents the average of the walking foot force characteristics of the 18 participants).

#### 3.2.2 Characteristics of foot functional units during running

**FIGURE 5:**
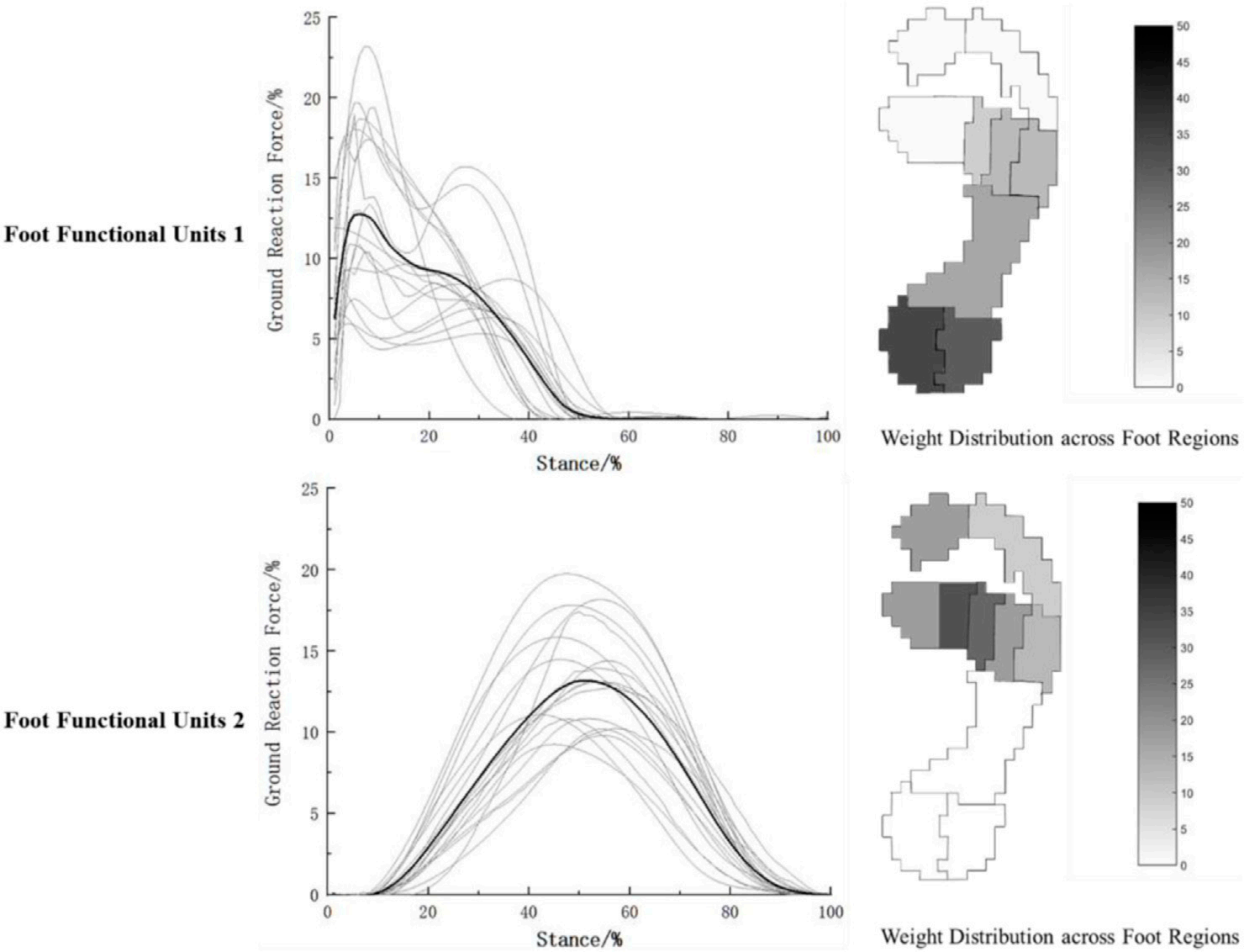
Characteristics of foot functional units during running.

NNMF was performed on the foot force characteristics during running, as shown in Figure 5. Similar to walking, the foot force characteristics during running were dominated by the heel and the second and third metatarsal bones. The overall force characteristics of the two foot functional units were concentrated respectively prior to 40% and between 40% and 60% of the stance phase during running.

(The gray lines show the foot force characteristics of each participant during running, and the black line represents the average of the running foot force characteristics of the 18 participants).

### 3.3 Comparison of foot functional units between walking and running

#### 3.3.1 Comparison of coefficient matrices between walking and running foot functional units

**FIGURE 6:**
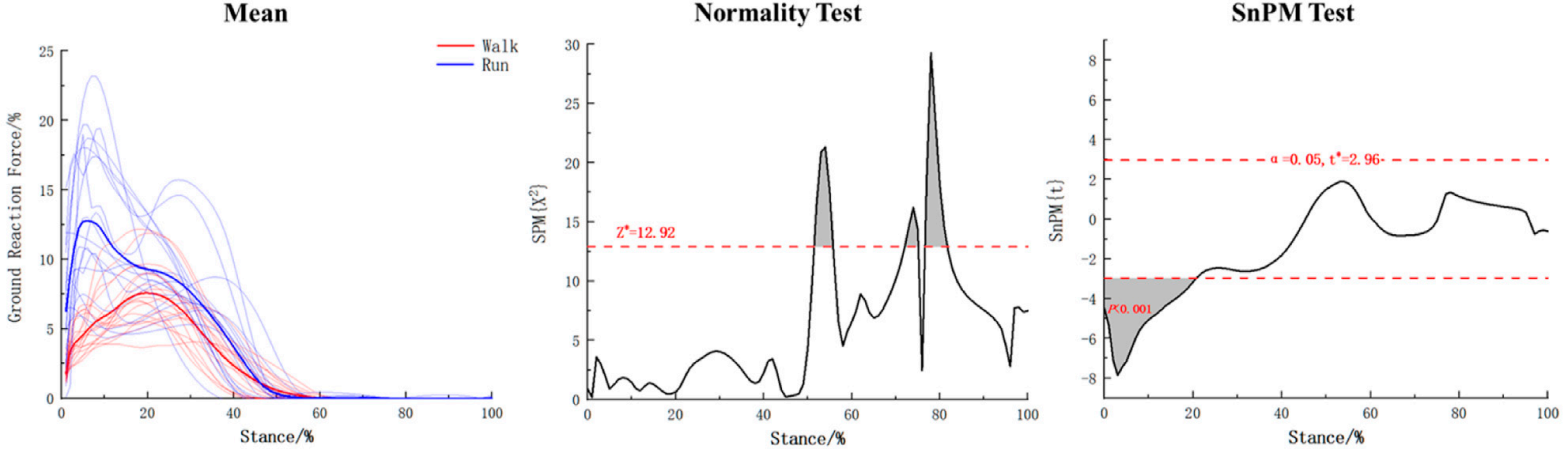
The coefficient matrix statistical results for walking and running in foot functional unit 1.

According to Figure 6, the coefficient matrix of walking and running in foot functional unit 1 does not follow a normal distribution. Therefore, a one-dimensional data test was performed using SnPM. The results showed that there was a significant difference (*p* < 0.001) in the coefficient matrix between walking and running during the 0%–20% stance phase in foot functional unit 1, with the overall force feature being higher during running than during walking in this phase.

**FIGURE 7:**
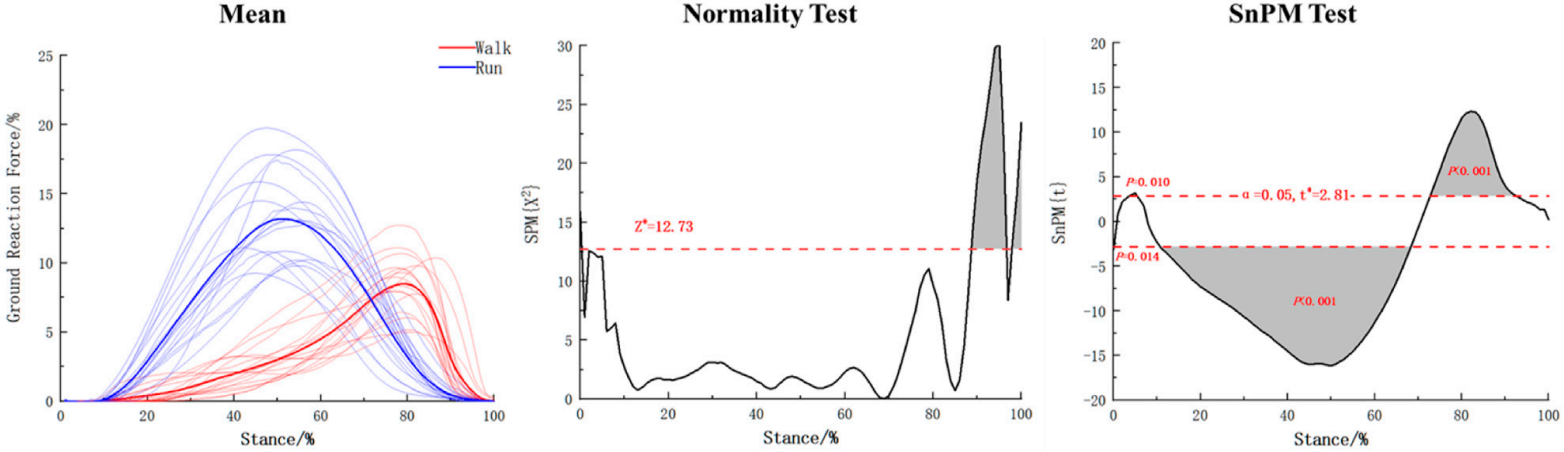
The coefficient matrix statistical results for walking and running in foot functional unit 2.

Based on Figure 7, it is evident that the overall force characteristics of walking and running in foot function unit 2 do not follow a normal distribution. Therefore, a one-dimensional data test using SnPM was conducted. It was found that the thresholds were exceeded at the beginning of the stance phase, 4%–5%, 11%–69%, and 73%–92% stages during walking and running. Specifically, the overall force during walking was lower than that during running at the beginning (*p* = 0.014) and 11%–69% stages (*p* < 0.001), while the overall force during walking was higher than that during running at the 4%–5% (*p* = 0.001) and 73%–92% stages (*p* < 0.001).

#### 3.3.2 Comparison of basic matrices between walking and running foot functional units

Table 1 presents the comparison results of the base matrices between walking and running in foot functional unit 1. It can be observed that the weights of M3, M4, and HL regions follow a normal distribution, and paired sample t-tests were performed, while the weights of other foot regions were examined using non-parametric tests. The results indicated that in foot functional unit 1, compared to walking, running had higher weight contributions to the total force from M3(*p* = 0.025) and M4 regions (*p* = 0.004), while lower weight contribution from HL region (*p* = 0.005).

**TABLE 1:** Comparison of basic matrices for walking and running in foot function unit 1 (n = 18%).

Foot regions	Walk		Run		*p-value*
	Mean/Median	Standard deviation/Interquartile range	Mean/Median	Standard deviation/Interquartile range	
T1	0.00	(0.00.0.00)	0.00	(0.00.0.00)	1.000
T2-5	0.00	(0.00.0.00)	0.00	(0.00.0.00)	0.317
M1	0.00	(0.00.0.00)	0.00	(0.00.0.00)	0.593
M2	0.00	(0.00.0.376)	0.00	(0.00.0.715)	0.424
M3	**1.21**	(**0.00**,**2.24**)	**1.47**	(**0.00**,**3.60**)	**0.025**
M4	**2.87**	**1.84**	**4.56**	**2.71**	**0.004**
M5	3.00	(1.66.4.84)	4.02	(2.13.7.05)	0.170
MF	12.00	(7.20.18.70)	11.88	(8.56.20.95)	0.215
HM	41.01	7.68	40.57	10.22	0.829
HL	**37.08**	**1.75**	**30.21**	**8.31**	**0.005**

Bold values indicate *p* < 0.05.

According to Table 2, in foot functional unit 2, the weights of M2, M3, and M4 areas conform to normal distribution, and paired sample t-tests were used for analysis, while Wilcoxon signed-rank test was used for the other foot regions. The results show that in the T1(*p* = 0.020), M1(*p* = 0.006), and MF(*p* = 0.017) regions, the contribution of total force during running was higher than walking, while in M3(*p* < 0.001) and M4(*p* = 0.004) regions, the weights of both areas for total force during running were lower than walking.

**TABLE 2:** Comparison of basic matrices for walking and running in foot function unit 2 (n = 18,%).

Foot regions	Walk		Run		*p-value*
	Mean/Median	Standard deviation/Interquartile range	Mean/Median	Standard deviation/Interquartile range	
T1	**7.78**	(**5.16,11.96**)	**15.06**	(**6.94**,**16.78**)	**0.020**
T2-5	2.05	(1.06.4.03)	3.94	(1.41.5.10)	0.215
M1	**9.82**	(**4.31**,**20.77**)	**15.45**	(**10.43,25.33**)	**0.006**
M2	26.78	9.35	27.07	7.08	0.842
M3	**27.12**	**8.05**	**21.34**	**6.53**	**<0.001**
M4	**14.37**	**8.08**	**9.97**	**3.67**	**0.004**
M5	4.23	(1.59.9.39)	4.27	(1.60.7.17)	0.744
MF	**0.00**	(**0.00,0.23**)	**0.60**	(**0.03,1.60**)	**0.017**
HM	0.00	(0.00.0.00)	0.00	(0.00.0.00)	1.000
HL	0.00	(0.00.0.00)	0.00	(0.00.0.00)	1.000

Bold values indicate *p* < 0.05.

## 4 Discussion

### 4.1 Feasibility analysis of non-negative matrix factorization in foot mechanics applications

Walking and rearfoot striking running, as basic movement patterns, exhibit different plantar foot mechanics during different phases of the support period. In traditional biomechanical analyses of human movement, peak forces, impulses, and loading rates are typically used to analyze foot-ground interactions. This method can provide detailed explanations of the mechanical changes in different regions, However, it cannot take into account the spatiotemporal characteristics and is unable to simplify the plantar area division from a functional perspective based on mechanical features. NNMF as an important feature extraction method can not only extract features from multidimensional data but also preserve the temporal and spatial variations in the data (Lee and Seung, 1999; Pataky et al., 2013; Nishida et al., 2017). Due to the non-negative characteristics of plantar pressure, NNMF can be used for simplified analysis of multiple regions of the foot. Van et al. used NNMF to perform functional segmentation of the foot based on plantar pressure characteristics, and found that this method had good discrimination for different types of running foot strikes (Van Hese et al., 2021). However, their study did not provide a specific quantitative analysis of the decomposed matrices. After applying NNMF to one-dimensional biomechanical data, a coefficient matrix and corresponding basis matrix are obtained. The coefficient matrix reflects the overall spatiotemporal features of the data in the form of a curve, while the basis matrix shows the weight values of each element in the matrix (Lee and Seung, 1999). In the analysis of walking and running plantar pressure, traditional t-tests can be used to compare the statistical differences in the contribution of each foot region based on the basis matrix. However, traditional hypothesis testing methods cannot reflect the continuous change characteristics of the overall force curve for the coefficient matrix. The statistical parameter mapping based on the theory of random fields has been used for the analysis of continuous data, including kinematic and kinetic data (Pataky, 2012; Pataky et al., 2015). In this study, we applied this method to compare the coefficient matrices obtained from NNMF of walking and running data, in order to achieve quantitative analysis of the decomposed matrices. Our results showed that under both walking and rearfoot striking running, when the number of matrix factorization was set to 2, the fitting degree of the reconstructed matrices exceeded the VAF value of 0.85 set in this study. This indicates that the dimensionality reduction analysis of rearfoot striking running and walking can be simplified into two dynamic foot functional units.

### 4.2 Analysis of foot functional unit features during walking and running

Our study used NNMF to divide the two foot functional units based on the main force application time of the overall foot mechanics during walking and running. During walking, the first foot functional unit mainly exhibited mechanical characteristics in the support phase around 15%–35%, at this point, dorsiflexion of the foot and flexion of the knee joint are used to complete the deceleration of the body, which is the weight-bearing buffering stage of walking (Earls, 2018; Perry and Burnfield, 2018). According to Figure 4 and Table 1, the HM and HL regions occupied 78.09% of the weight of all foot regions, indicating that the heel region played a major role in buffering during weight-bearing, while the midfoot (12%) and M3-M5 regions (7.08%) also participated in buffering. This suggests that the heel receives the highest impact during weight-bearing buffering, and the arch and outer edge of the forefoot assist in completing the buffering. In the second foot functional unit during walking, as shown in Figure 4 and Table 2, the main force is concentrated around the support phase of 60%–80%, during this phase, the posterior muscles of the lower leg and the muscles of the foot plantar flex to perform push-off, while the muscles of the thigh and buttocks begin to exert force, extending the knee joint and propelling the body forward (Earls, 2018; Perry and Burnfield, 2018). The basic matrix shows that the M2-M4 region occupies 68.27% of the weight, indicating that the middle part of the forefoot is the main force application area during push-off. Early studies on foot mechanics during walking typically considered the foot to be supported by three points: the heel, lateral forefoot, and medial forefoot (Zheng, 2002). With advances in foot pressure testing methods, researchers found that the results of actual walking tests contradicted the three-point support theory. This may be due to traditional understanding not taking into account the mechanical characteristics of the metatarsal bone section (Zheng, 2002). Kanatli's research discovered that in a healthy population, the middle area of the forefoot bears the highest pressure during walking (Kanatli et al., 2008). This finding aligns with the our results, which show that the primary force regions during the support phase of walking are the heel and middle forefoot. In contrast to previous studies, our study provides a more detailed functional division based on foot mechanics, namely, that the heel is the main force area during the buffering phase of walking and the middle forefoot is the main force area during the push-off phase.

During running, the plantar mechanical characteristics are similar to walking. In the first functional unit, the main force is concentrated around 10%–30%, with the heel being the main force area (70.78%), and the midfoot (11.88%) and the medial-lateral forefoot (10.05%) assisting in cushioning, meanwhile, the hip joint, knee joint, and ankle joint sequentially flex to store kinetic and gravitational potential energy as elastic potential energy (Mcmahon and Cheng, 1990). In the second functional unit, as shown in Figure 5 and Table 2, the main force characteristics are concentrated around 40%–60% of the running support phase, with the M1, M2, and M3 regions accounting for 63.86% of the weight and the T1 region accounting for 15.06% of the weight. It can be seen that during the push-off phase of running, the main force areas are the medial and central parts of the forefoot and the toes, and the foot exhibits an outward and rotational posture during force exertion, gradually shifting the force from the lateral to the medial side of the foot. Venkadesan’s team found that the transverse arch of foot provides over 40% of the foot’s stiffness through variations in the arrangement of the metatarsal bones (Venkadesan et al., 2020). During the push-off phase of running, the metatarsophalangeal joint undergoes flexion to increase the tension of the plantar fascia, enhancing foot stiffness through the “windlass effect”. Subsequently, the metatarsophalangeal joint extends, releasing stored elastic potential energy to improve the efficiency of push-off (Donatelli, 1987; Kelly et al., 2015). The significant role of the metatarsophalangeal joint in this process may be the reason for the prominent mechanical characteristics in the forefoot region observed in this study. At the same time, the results of this study show that, similar to walking, the forefoot area still plays a role in the buffering functional unit during rearfoot strike running. During the buffering phase of walking or rearfoot strike running, the foot plantarflexes around the heel axis and the forefoot assists in weight-bearing (Perry and Burnfield, 2018); after the heel lifts off, the foot rolls around the toe axis, and at this point, the forefoot completes the push-off phase (Perry and Burnfield, 2018). In these two processes, the transverse arch of the forefoot participates in both buffering and push-off, indicating its important role in the transition from elastic buffering to rigid lever function. However, since this study only investigated the foot’s mechanical characteristics, further research on the arch should incorporate morphological changes in the foot during the support phase for deeper insights.

### 4.3 Comparison analysis of foot function units between walking and running

Quantitative analysis of the basic matrix and coefficient matrix within the two functional units of walking and running was conducted in this study. Results showed that in the first functional unit, as shown in Figure 6 and Table 1, during the first 20% of the support period, the overall force characteristics of the foot were higher in running than in walking (*p* < 0.001). This study also found that in this functional unit, the contribution weights of the M3 (*p* = 0.025) and M4 (*p* = 0.004) regions were higher in running than in walking, while the weight of the HL region was lower in running than in walking (*p* = 0.005), indicating that the foot experiences greater impact during running in the first 20% of the support period, and that compared to walking, the weight of the lateral part of the heel is smaller during the buffering phase of rearfoot landing when running, with the weight-bearing area more biased towards the forefoot, suggesting that the forefoot may be more involved in buffering under high loads. In the second functional unit, as shown in Figure 7 and Table 2, it was found that at the beginning of the support period, the overall force characteristics of walking were lower than those of rearfoot running (*p* = 0.014). During the 4%–5% support phase, walking was higher than rearfoot landing when running (*p* = 0.010), due to these two phases are short in duration, they cannot reflect curve characteristics and are not further analyzed. During the support phase from 11% to 69%, the overall force characteristics of walking were lower than running (*p* < 0.001), while during the support phase from 73% to 92%, the overall force of walking was higher than that of running (*p* < 0.001). Combined with Figure 7, it can be seen that in the push-off unit, running completed the main force production process earlier than walking. For the basic matrix, the results of this study found that in the T1 (*p* = 0.020), M1 (*p* = 0.006), and MF (*p* = 0.017) areas, the weight of the heel strike during running was higher than that during walking, while in the M3 (*p* < 0.001) and M4 (*p* = 0.004) areas, the weight of running was lower than that of walking. This indicates that running places greater stress on the arch area of the foot during the heel strike phase compared to walking. Moreover, in the forefoot area, the force production area during running shifts from the lateral to the medial side compared to walking, which allows for more complete toe-off extension. Wang and Raychoudhury found that during running, the functional axis of the metatarsophalangeal joint moved forward by about 3% compared to walking (Raychoudhury et al., 2014; Wang et al., 2021), which may have contributed to the greater contribution of the toe region during running in this study.

There are also some limitations in this study. Although the quality of NNMF for both walking and running in this study reached 0.95, there still exists some error between the factorization and the real data. Future research could increase the number of matrix factorizations according to different needs to improve the quality of the reconstructed matrix. In addition, our study used NNMF to divide the plantar into ten regions and analyzed the differences in plantar mechanical characteristics between healthy individuals during walking and running. However, some studies have shown that individuals with symptoms, such as stroke, pes cavus, and flatfoot, exhibit differences in plantar mechanics compared to healthy individuals during walking (Hillstrom et al., 2013; Wang et al., 2019; Bai et al., 2022). Therefore, it is recommended to redefine the plantar regions and combine lower limb biomechanical features for functional classification when applying NNMF to symptomatic populations based on their movement patterns. Additionally, NNMF can be used to further analyze foot function from a mechanical perspective for different types of movements, such as cutting and sudden stops, in healthy individuals. Future research should increase sample sizes and apply NNMF to functional analysis in different populations and complex movements from a mechanical perspective.

## 5 Conclusion

In this study, NNMF was used to decompose walking and running into two foot functional units based on mechanical characteristics. According to the matrix structure of each unit, the two foot functional units corresponded to cushioning and push-off, respectively. The forefoot accounted for a certain weight in both cushioning and push-off functions, indicating the existence of complex foot functional transformation mechanisms in the transverse arch of foot. Compared with walking, running completed push-off earlier, with a force application area that was more inwardly biased towards the foot and with the big toe accounting for a larger weight during push-off. The use of NNMF to extract and quantify foot mechanics has certain application value.

## Data Availability

The original contributions presented in the study are included in the article/Supplementary material, further inquiries can be directed to the corresponding author.
